# Effects of TiO_2_ and Co_3_O_4_ Nanoparticles on Circulating Angiogenic Cells

**DOI:** 10.1371/journal.pone.0119310

**Published:** 2015-03-24

**Authors:** Valentina Spigoni, Monia Cito, Rossella Alinovi, Silvana Pinelli, Giovanni Passeri, Ivana Zavaroni, Matteo Goldoni, Marco Campanini, Irene Aliatis, Antonio Mutti, Riccardo C. Bonadonna, Alessandra Dei Cas

**Affiliations:** 1 Department of Clinical and Experimental Medicine. Division of Endocrinology. University of Parma and Azienda Ospedaliero-Universitaria of Parma, Parma, Italy; 2 Department of Clinical and Experimental Medicine. Unit of Occupational and Environmental Medicine. University of Parma, Parma, Italy; 3 Department of Clinical and Experimental Medicine. Unit of Andrology, Metabolic Bone Diseases and Endocrinology. University of Parma, Parma, Italy; 4 Department of Clinical and Experimental Medicine. Unit of Diabetes and prevention of associated diseases. University of Parma, Parma, Italy; 5 IMEM-CNR (Istituto Materiale per l’Elettronica ed il Magnetismo – Consiglio Nazionale delle Ricerche) Institute, Parma, Italy; 6 Department of Physics and Earth Sciences. University of Parma, Parma, Italy; University of Sassari, ITALY

## Abstract

**Background and Aim:**

Sparse evidence suggests a possible link between exposure to airborne nanoparticles (NPs) and cardiovascular (CV) risk, perhaps through mechanisms involving oxidative stress and inflammation. We assessed the effects of TiO_2_ and Co_3_O_4_ NPs in human circulating angiogenic cells (CACs), which take part in vascular endothelium repair/replacement.

**Methods:**

CACs were isolated from healthy donors’ buffy coats after culturing lymphomonocytes on fibronectin-coated dishes in endothelial medium for 7 days. CACs were pre-incubated with increasing concentration of TiO_2_ and Co_3_O_4_ (from 1 to 100 μg/ml) to test the effects of NP – characterized by Transmission Electron Microscopy – on CAC viability, apoptosis (caspase 3/7 activation), function (fibronectin adhesion assay), oxidative stress and inflammatory cytokine gene expression.

**Results:**

Neither oxidative stress nor cell death were associated with exposure to TiO_2_ NP (except at the highest concentration tested), which, however, induced a higher pro-inflammatory effect compared to Co_3_O_4_ NPs (p<0.01). Exposure to Co_3_O_4_ NPs significantly reduced cell viability (p<0.01) and increased caspase activity (p<0.01), lipid peroxidation end-products (p<0.05) and pro-inflammatory cytokine gene expression (p<0.05 or lower). Notably, CAC functional activity was impaired after exposure to both TiO_2_ (p<0.05 or lower) and Co_3_O_4_ (p<0.01) NPs.

**Conclusions:**

In vitro exposure to TiO_2_ and Co_3_O_4_ NPs exerts detrimental effects on CAC viability and function, possibly mediated by accelerated apoptosis, increased oxidant stress (Co_3_O_4_ NPs only) and enhancement of inflammatory pathways (both TiO_2_ and Co_3_O_4_ NPs). Such adverse effects may be relevant for a potential role of exposure to TiO_2_ and Co_3_O_4_ NPs in enhancing CV risk in humans.

## Introduction

Nano-sized particles undergo wide industrial application by virtue of their peculiar physicochemical properties [1]. Among nanoparticles (NPs), titanium dioxide (TiO_2_) is widely used as photocatalyst in sunscreens, cosmetics, paints, plastics, paper, pharmaceutical additives, food colorants; whereas cobalt oxide (Co_3_O_4_) is often employed as a pigment, a catalyst, and a sensor in electrochemistry, magnetism and energy storage [2,3].

In light of their increasing use, concerns about NP effects on human health are not limited to occupational settings, but also include consumer exposure. Physicochemical properties of nanosized materials such as particle size, shape (spherical, fiber, crystal), coating, morphology and agglomeration capacity, may play a role in determining potential harmful health effects of NP-based materials [4,5].

Environmental exposure to combustion-derived particulate matter (PM) has been associated with progression of atherosclerosis, myocardial infarction and CV mortality in humans [6,7], the nanosized fraction of PM being considered a potential culprit [8]. Pulmonary instillation of TiO_2_ NPs results into plaque progression in atherosclerosis-prone apolipoprotein E-deficient (apoE^−/−^) mice [9].

There is evidence that ambient exposure to NPs results into direct interaction between NPs and several potential target cells. Due to their small size NPs can enter the circulatory system through multiple routes, including inhalation [10], ingestion and transdermal absorption [11], and their presence has been detected in distant organs (i.e. kidney and liver) [12].

In humans, studies addressing TiO_2_ and Co_3_O_4_ effects on the CV system are limited by the difficulty to quantify NP exposure and by ethical issues. Thus, mechanistic studies are to be performed in human cells involved in the maintenance of CV homeostasis. Endothelial cells represent potential targets for NP toxicity in humans and NP translocation into endothelial cells may lead to endothelial dysfunction [13,14]. In human umbilical vein endothelial cells (HUVEC) [15] and in primary vascular endothelial cells [16], TiO_2_ NPs increased oxidative stress, activated inflammatory pathways and promoted the expression of adhesion molecules. Similarly, Co_3_O_4_ NPs impaired cell viability and induced oxidative stress in a line of endothelial-like cells [17].

Endothelial health and function *in situ* reflect the balance between the extent of cell injury and the endogenous endothelial repairing capacity [18]. This repair/replacement process is mediated also by endothelial progenitor cells (EPCs), a heterogeneous cell population including also cells of myeloid origin named circulating angiogenic cells (CACs), which substantially contribute to vascular homeostasis [19]. CAC number and function have been consistently shown to be altered in relation to the presence of CV risk factors and disease [20,21] providing clinical information regarding the atherosclerotic burden and the future CV risk [22]. NPs may be detrimental to vascular health *in vivo* not only by inducing direct dysfunction/damage of endothelium, but also by hampering endothelial repairing processes. To the best of our knowledge, the effects of NPs in human EPCs have never been investigated.

We therefore tested *in vitro* the following hypotheses: a) NPs can impair human CAC viability and/or function; b) they may do so by promoting apoptosis, oxidative stress and inflammation.

## Methods

### Ethics Statement

The study protocol was in accordance with the Declaration of Helsinki and was approved by the local Institutional Review Board (*Comitato Etico Unico della Provincia di Parma*). The need for informed consent was waived due to the fact that blood donor material used was fully anonymized.

### Circulating angiogenic cell isolation

CACs were isolated, according to literature [19] as previously described [23,24]. Briefly, peripheral blood mononuclear cells (PBMCs) of healthy donor buffy-coats were isolated by Lymphoprep (Euroclone, Milano, Italy) density gradient centrifugation. A total of 10^7^ cells/well were seeded into fibronectin-coated six-well culture plates and cultured in endothelial cell growth medium-2 (EGM-2) (Lonza, Milano, Italy) with supplements in 5% CO_2_ at 37°C. On day 7, adherent cells displaying a spindle-shaped morphology were considered CACs. CACs were pre-incubated with increasing working concentrations (1–10–20–50–100 μg/ml) of TiO_2_ and Co_3_O_4_ NPs or with vehicle (bovine serum albumin, BSA) as negative control.

### Particle suspensions

TiO_2_ and Co_3_O_4_ powders (677469 and 637025 from Sigma-Aldrich respectively) were dispersed and sonicated (power consumption: 7W, 1ml dispersion, 60 seconds sonication) (Heat Systems Ultrasonics Inc., Farmingdale, NY, USA) in distilled water and stabilized with BSA 1.5 mg/ml (Sigma Aldrich, St Louis, Missouri, USA) [25] to mimic circulating nanoparticle-protein complexes. Stock solutions (kept in the dark by wrapping them in aluminum foil) were obtained by diluting samples in phosphate buffered saline (PBS from Euroclone) and dissolved in fresh EGM-2 medium after sonication for 1 minute immediately before use.

## Characterization of TiO_2_ and Co_3_O_4_ nanoparticles

Detailed methods on nanoparticle characterization are reported in S1 Text.

### Structural and morphological characterization

Structural and morphological characterization of NPs were performed by Transmission Electron Microscopy (TEM) on a 200 kV analytical JEOL JEM 2200-FS and subsequently by both Dynamic Light Scattering (DLS) and Z-potential techniques (90Plus PALS instrument by Brookhaven Corporation). At TEM analyses TiO_2_ NPs showed a regular spherical shape and appear as slightly aggregated, whereas Co_3_O_4_ NPs revealed an irregular non spherical shape, with tendency to form agglomerates of tens of NPs [26]. Z-potential values, reported in Table 1, showed that the effect of EGM-2 is to reduce the surface charge of the NPs, probably due to the coverage of NPs by charged molecules present in the medium.

**Table 1:** Z-potential values of Co_3_O_4_ and TiO_2_ NPs measured in different dispersion liquids.

	**H_2_O**	**PBS-BSA**	**EGM2**
**Co_3_O_4_**	mean: −19.08 mV; SD: 1.15	mean: −7.85 mV; SD: 1.97	mean: −4.7 mV; SD: 3.57
**TiO_2_**	mean: −31.74 mV; SD: 1.02	mean: −13.05 mV; SD: 2.13	mean: +9.8 mV; SD: 2.44

PBS = phosphate buffered saline; BSA = bovine serum albumin; EGM-2 = endothelial cell growth medium; SD = standard deviation.

### Determination of anatase and rutile proportion in TiO_2_ powder.

Commercial TiO_2_ NP products are a mixture of different TiO_2_ polymorphs; in this study TiO_2_ NP features were assessed by Raman spectroscopy. Raman spectra collected on the TiO_2_ NPs displayed peaks corresponding to a mixture of anatase and rutile [27]. The results of fitting curves indicated 93±1% anatase in the TiO_2_ powder.

### Viability assay

The effect of increasing working TiO_2_ and Co_3_O_4_ NP concentrations for 24 and 48 h was tested on CAC viability by VisionBlue fluorescence cell viability assay kit (Biovision, Mountain View, CA) following manufacturer's instructions and as previously described [23,24]. Four independent experiments were performed in twelve replicate wells for each exposure time and condition. Results are expressed as amount of cells normalized to BSA control values (vehicle). Based on results from cell viability assay, which showed a comparable effect at 24 and 48 h, we selected the 24 h time-point as the exposure time to be used in each experimental test.

### Fibronectin Adhesion assay

Fibronectin cell adhesion assay was used to test the effects of TiO_2_ and Co_3_O_4_ NPs on CAC function. CACs were exposed to increasing concentrations (1–20–100 μg/ml) of TiO_2_ and Co_3_O_4_ NPs for 24 h. Following detachment with Trypsin-EDTA (Sigma-Aldrich), cells were seeded into fibronectin-coated 24-well culture plates at the final density of 3x10^4^ viable cells/well and incubated at 37°C for 3h. Adherent cells were washed twice with PBS and counted in six random fields for each culture condition using a phase-contrast microscopy (Leica DMIL, Wetzlar, Germany). Four independent experiments were performed and the number of adherent cells was normalized to BSA control values.

### Caspase 3/7 activity

Caspase-Glo 3/7 assay was used to investigate pro-apoptotic effects of increasing working concentrations of TiO_2_ and Co_3_O_4_ NPs, according to manufacturer’s instructions (Promega Corporation, Madison, WI, USA). CACs were cultured in 96-well culture plate (2.5x10^5^ cells/well) and exposed to TiO_2_ and Co_3_O_4_ NPs for 24 h. Cells were then washed with PBS and incubated with 100 μl of Caspase-Glo 3/7 reagent at 37°C for 30 min. Luminescence was measured by Cary Eclipse fluorescence spectrophotometer (Varian/Agilent, Santa Clara, CA, USA). Staurosporine (100 nM for 24h) was used as a positive control of apoptosis. Three independent experiments were performed and each sample was measured in six replicates. Fold increase in caspase activity was normalized to the activity obtained from BSA-treated cells.

### Lipid peroxidation products

Lipid peroxidation was evaluated by ‘‘Thiobarbituric Acid Reactive Substances” (TBARS) method, as previously described [28], using Malondialdehyde as a standard for the calibration curve (range 0–10 μM). In preliminary experiments, we documented that cells treated with H_2_O_2_ 50μM for 30 min displayed substantial increases (about 2-fold) in TBARS when compared to untreated cells, thereby validating the use of TBARS as a “footprint” of increased oxidative stress in our experimental setting.. The fluorescence intensity was measured by Cary Eclipse fluorescence spectrophotometer (Varian/Agilent) (excitation 515 nm, emission 545 nm). A concentration of 20μg/ml of TiO_2_ and Co_3_O_4_ NPs was selected based on IC_50_ (inhibitory concentration 50%) values from cell viability assay. No other concentrations were tested due to cell number limit (at least 10^6^ CACs were needed to perform this assay). Three independent experiments were carried out and TBARS values were normalized to protein concentrations and expressed as percentage of control (BSA).

### Pro-inflammatory cytokine/chemokine gene expression

The effect of TiO_2_ and Co_3_O_4_ NPs in modulating CAC pro-inflammatory response was assessed by gene expression assay. Cells were lysed with QIAzol lysis reagent (Qiagen Ltd, West Sussex, UK) and total RNA was extracted using miRNeasy Mini Kit (Qiagen) and quantified (NanoDrop-NanoDrop Technologies, DE). Starting from 300 ng of total RNA, iScript Reverse Transcription Kit (Bio-Rad Laboratories, Inc. Hercules, Ca, USA) was used to obtain cDNA in accordance with manufacturer’s instructions. Reactions without reverse transcriptase were performed in parallel as a negative control. Interleukin (IL)-1β, tumor necrosis factor-α (TNF-α), and monocyte chemoattractant protein-1 (MCP-1) gene expression was assessed by qPCR as already reported [23] using iTaq Universal Probes Supermix (Bio-Rad) with TaqMan primers and probe (Applied Biosystems, Carlsbad, CA, USA) on a CFX Connect Real-Time (Bio-Rad). Specific thermal cycling conditions were used: 98°C for 30 sec, followed by 40 amplification cycles (95°C for 3 s; 60°C for 20 sec). In preliminary experiments, we documented that cells treated with LPS 100 nM displayed manifold increases in IL-1β, TNF-α, and MCP. Seven independent experiments were performed and samples were analyzed in triplicate. Gene expression values were calculated based on the ΔΔCt method and the relative level of expression was calculated using human GAPDH as reference gene. In addition to 24 h, we assessed inflammatory response also at 6 h, which according to our experience and the literature [29], is associated with a peak in inflammation following different cues.

### Statistical analysis

Data were expressed as mean±SD (standard deviation) of at least three independent experiments. Two-way ANOVA followed by Dunnet *post-hoc* test was performed to detect the differences among different culture conditions, using the NP concentration as fixed factor and the number of experiment as random factor to take into account the replicates per experiment. In gene expression assay, data were not normally distributed; therefore the Kruskal-Wallis ANOVA followed by Dunn’s *post-hoc* test was used to compare culture conditions. Statistical significance was set at p<0.05 (two-sided). Data analysis was performed using SPSS version 20.0 (SPSS Inc/IBM, Chicago, Ill, USA). Non-linear fitting to Hill function was performed with Origin 6.0 (Originlab, Northampton, MA). Specifically, the following function was used:

$$\text{Response}=\;{\text{V}}_{\text{MAX}}\left[1-\frac{{\left(\text{NP}\right)}^{\text{n}}}{{\text{IC}50}^{\text{n}}+{\left(\text{NP}\right)}^{\text{n}}}\right)$$

and each parameter (Vmax, n, IC50) was estimated with its SE by means of Levemberg-Marquardt algorithm.

## Results

### Effects of NPs on CAC morphology, viability and adhesion

**Fig 1:**
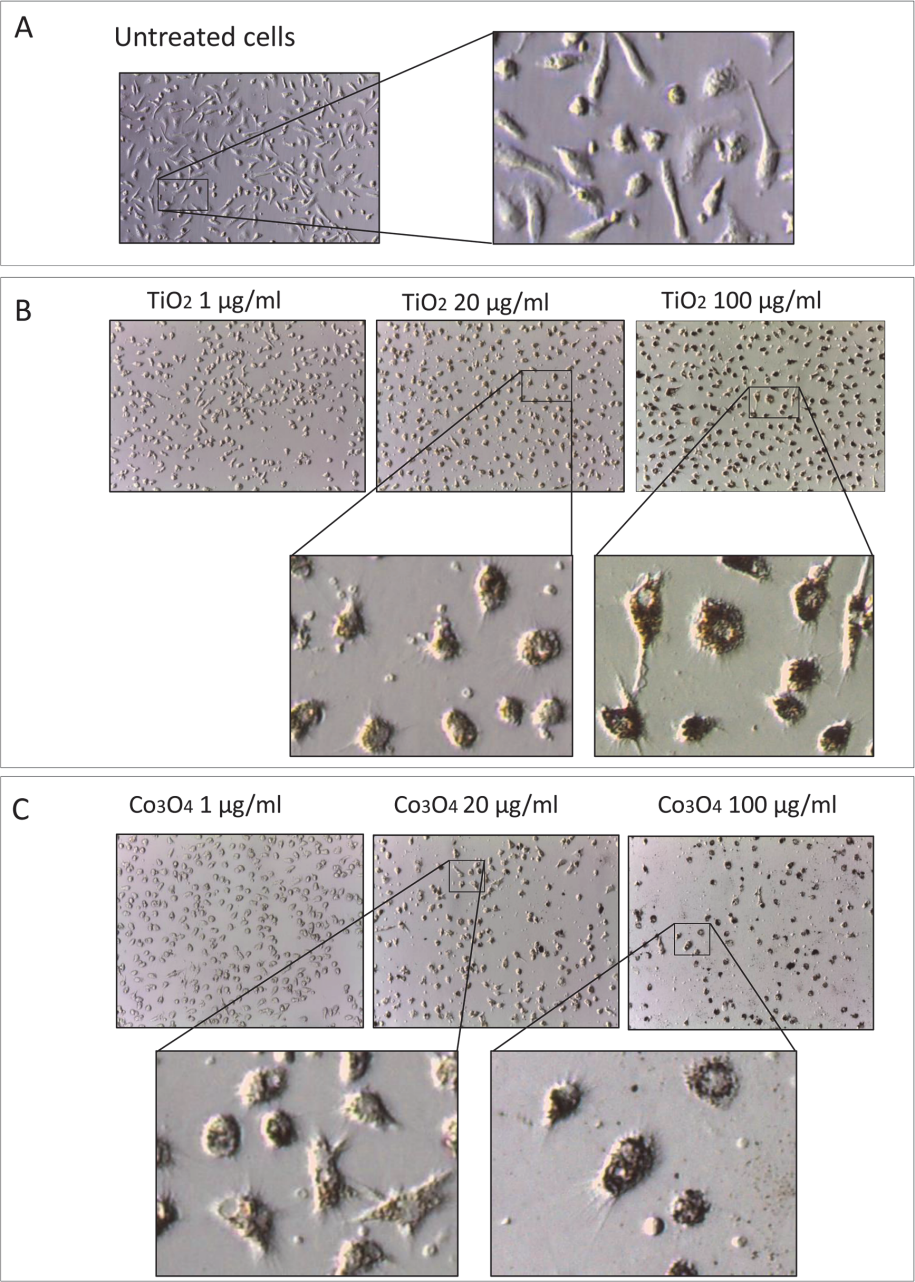
Cellular morphology. Morphology of CACs cells untreated (A) and following incubation with different concentration of TiO_2_ (B) and Co_3_O_4_ NPs (C). (CAC = circulating angiogenic cells).

CACs pre-incubated with NPs switched from an elongated to a spherical irregular shape (Fig. 1). Both TiO_2_ and Co_3_O_4_ NPs were internalized in cell cytoplasmic compartment in a concentration-dependent way.

**Fig 2:**
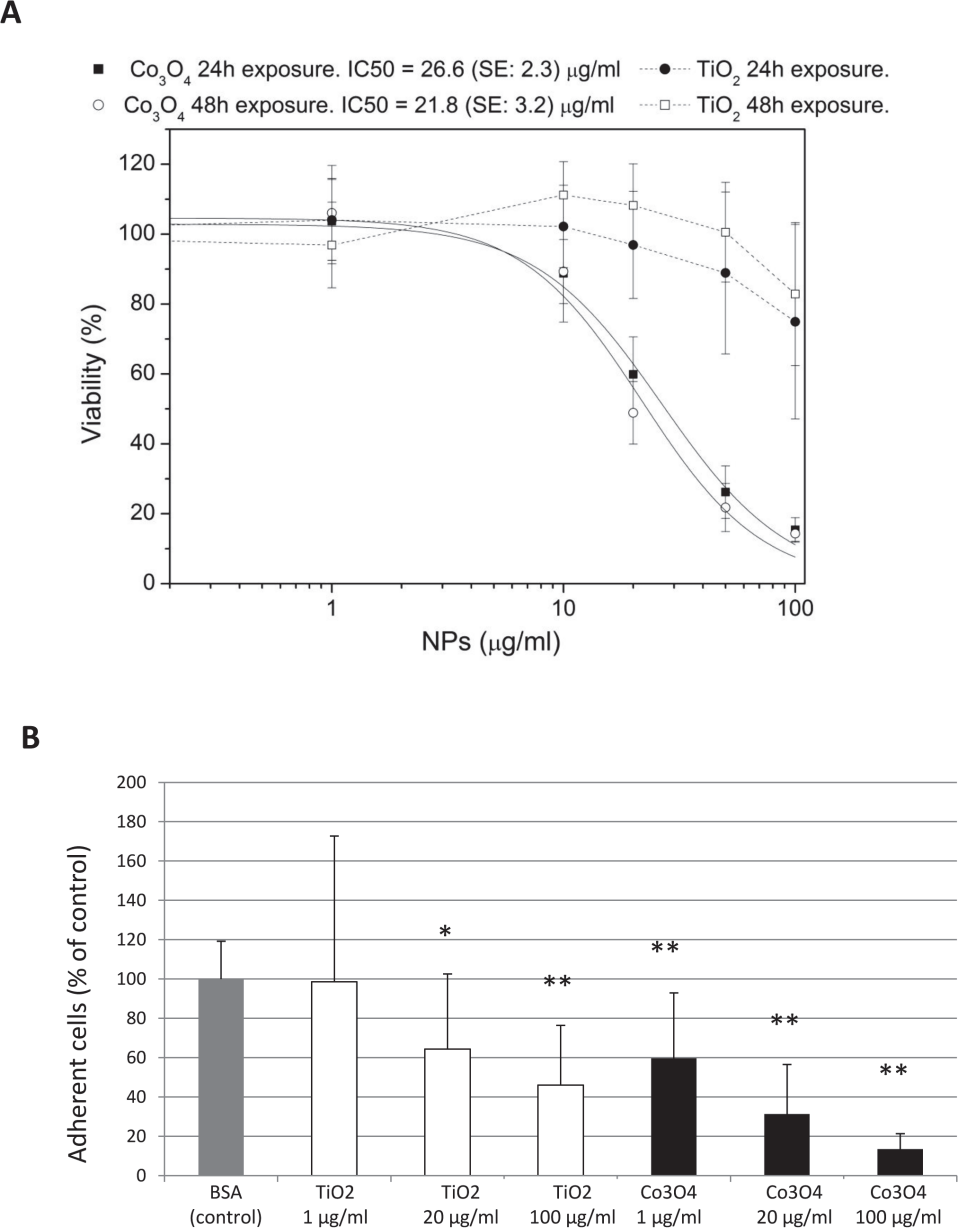
NP effects on CAC viability. Effect of TiO_2_ and Co_3_O_4_ NPs in affecting CAC viability following 24 and 48h of exposure (A) function assessed by adhesion assay on fibronectin (B) in CACs following 24h of exposure (NP = nanoparticle; BSA = bovine serum albumin; SE = standard error) (*p<0.05; **p<0.01 vs control).

A concentration-dependent reduction in cell viability was observed following both TiO_2_ and Co_3_O_4_ NP exposure, although Co_3_O_4_ elicited greater cytotoxic effect than TiO_2_. Specifically, after fitting the Co_3_O_4_ concentration-response curves with a Hill function, the IC_50_ was 26.6 μg/ml at 24 h and 21.8 μg/ml at 48 h, with substantially overlapping curves. Fitting the concentration-response curve by the Hill function and computing the IC_50_ was not possible for TiO_2_ NPs (Fig. 2A), because viability was still around 80% at the highest concentration tested (100 μg/ml).

Adhesion of CACs to fibronectin, an extra-cellular matrix protein component, was assessed as a readout of cell function. Incubation with either TiO_2_ or Co_3_O_4_ NPs impaired CAC adhesion to fibronectin in a concentration-dependent manner (Fig. 2B), with Co_3_O_4_ NPs showing a greater effect than TiO_2_.

### Effects of NPs on CAC apoptosis, oxidant stress and pro-inflammatory cytokine gene expression

**Fig 3:**
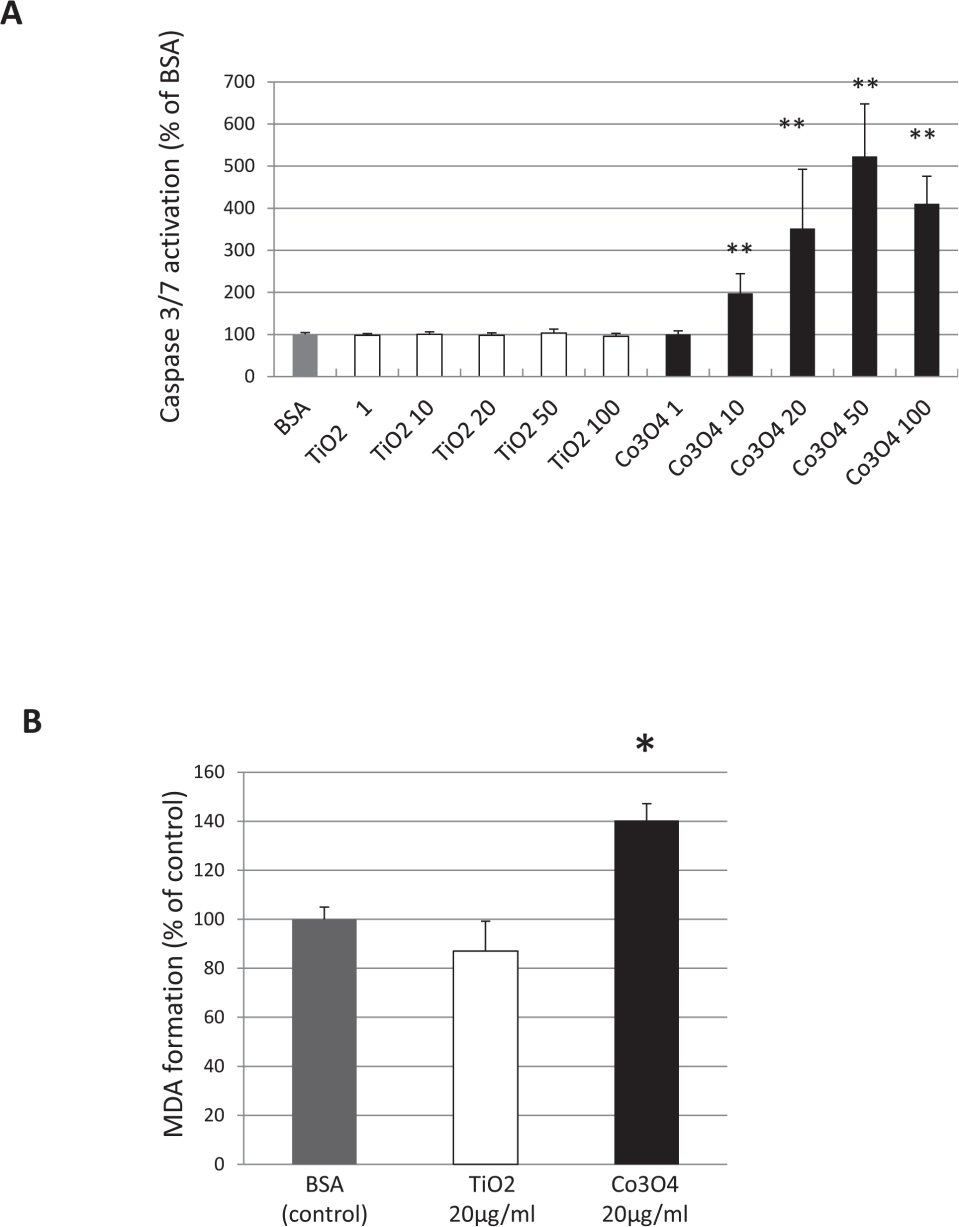
Effects of NPs on CAC apoptosis and oxidative stress. Effect of TiO_2_ and Co_3_O_4_ NPs on caspase 3/7 activation (A) and on oxidant stress formation (TBARS) (B) in CACs following 24 h of exposure (MDA = Malondialdehyde; NP = nanoparticle; BSA = bovine serum albumin) (*p<0.05 vs control; **p<0.01 vs control).

TiO_2_ and Co_3_O_4_ NP capacity to induce apoptosis was assessed by measuring the activation of Caspase 3/7, known to be the “effector” caspases. As shown in Fig. 3A, cells treated with Co_3_O_4_ NPs showed significantly increased caspase activity. The highest pro-apoptotic effect in CACs was observed at 50 μg/ml (p<0.001), but significant changes were detected already at 10 μg/ml (p<0.001). On the other hand, TiO_2_ NPs showed no significant pro-apoptotic action.

Induction of oxidative stress by TiO_2_ and Co_3_O_4_ (20 μg/ml) exposure for 24 h was assessed by measuring TBARS. The incubation with Co_3_O_4_ increased TBARS formation of about 40% in CACs compared to control (p<0.05) whereas TiO_2_ NP exposure showed no changes in peroxidation product formation (Fig. 3B).

**Fig 4:**
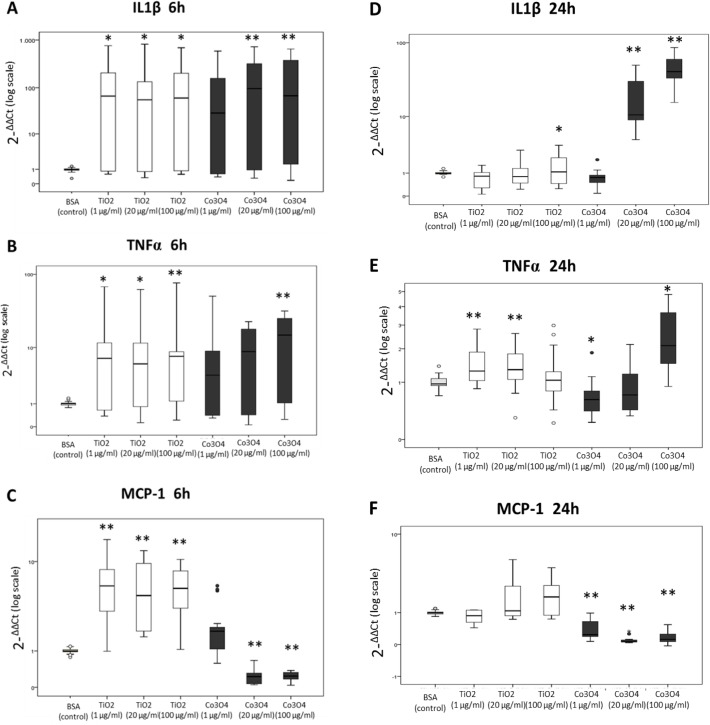
Effects of NPs on CAC inflammation. Effects of TiO_2_ and Co_3_O_4_ NPs on IL-1β (A), TNF-α (B) and MCP-1(C) gene expression in CACs following 6h and 24h (D-F) of exposure. Data are expressed as -ΔΔCt and represent the relative gene expression of CACs cultured in the presence of NPs in relation to control, normalized for the endogenous control GAPDH. (*N = 7*; *p<0.05 vs control;** p<0.01 vs control) (IL-1β = interleukin-1β; TNF-α = tumor necrosis factor-α; MCP-1 = monocyte chemoattractant protein-1; BSA = bovine serum albumin).

Incubation with TiO_2_ NPs significantly increased IL1-β, TNF-α and MCP-1 gene expression at each concentration tested, mainly at the early time point (6 h). The presence of Co_3_O_4_ in the culture medium also amplified the release of pro-inflammatory cytokines, although MCP-1 gene expression resulted highly repressed following Co_3_O_4_ NP incubation for 6 and 24 h (Fig. 4).

## Discussion

To the best of our knowledge, this is the first evidence that Co_3_O_4_ and, to a much lesser extent, TiO_2_ NPs can adversely affect *in vitro* human circulating angiogenic cells, which play a key role in vascular endothelial health [19,21]. Both Co_3_O_4_ and TiO_2_ particles were internalized by CACs and impaired CAC adhesion to fibronectin matrix, an obligate step *in vivo* for the process of vascular endothelial replacement and repair [30]. Furthermore, Co_3_O_4_, and to a much lesser extent also TiO_2_ NPs, exhibited an effect on CAC viability, consistently with evidence of *in vitro* cytotoxicity induced by Co_3_O_4_ [17,31,32], whereas no *in vivo* evidence is available to date.

The *in vivo* studies on TiO_2_ NP toxicity are controversial: inhalation or intra-tracheal instillation of TiO_2_ NPs induced pulmonary toxicity and inflammation in rats [33,34]. On the contrary, inhalation or intra-tracheal instillation of TiO_2_ NPs induced inflammation, but no genotoxic effects in mice [35] and in rats [36].

A key question is whether the *in vitro* concentrations of NP herein tested (1–100 ug/ml) are relevant to human exposure. Bioaccessibility and bioavailability *in vivo* are difficult to assess. However, it is known that ≈10–15% of inhaled TiO_2_ NPs can gain access to the vasculature [37]. Extrapolations based on this observation suggest that the NP concentrations tested in this study can be achieved in the bloodstream and in target organs of exposed subjects. Furthermore, NP concentrations used in this study were in line with, or even lower than, those used in previous works [15–17]. Therefore, the possible implications of the present study for risk assessment concerning environmental and especially occupational exposure are that Co_3_O_4_ and TiO_2_ NPs can impair endothelial homeostasis, via alterations in CAC function and viability, which ultimately may result into stunted endothelial repair and replacement. It should be noted that CAC impairment is considered a key mechanism contributing to adverse clinical CV end points and that circulating CACs are an independent predictor of CV morbidity and mortality [22]. Our findings may add biological support to the epidemiological observation that air pollution is associated with increased mortality. In addition to arrhythmogenic properties of combustion-based particles, accelerated atherosclerosis and increased CV morbidity have also been suggested as possible underlying mechanisms accounting for increased mortality in polluted cities [6–8]. CACs may be at least one of the primary cellular targets through which a detrimental effect of NPs on vascular health may be exerted. The novelty of our findings is that most of the available evidence is relevant to direct endothelial dysfunction/damage caused by NPs [15–17], whereas little or no attention has been devoted to the other key component of endothelial homeostasis, necessarily including the process of endothelial repair.

We also carried out exploratory studies to gauge the mechanism potentially engaged by NPs in altering CAC function and viability. To this aim we assessed selected readouts of apoptosis, oxidative stress and inflammation.

The activity of caspase 3/7, the final effectors of apoptosis, was greatly enhanced by Co_3_O_4_ NPs, but was not affected by TiO_2_ NP exposure. This is in agreement with the results of cell viability, and strongly suggests that apoptotic death is the primary mechanism elicited by Co_3_O_4_ NPs to reduce CAC viability in a dose-dependent manner. The small reduction in CAC viability at the highest TiO_2_ concentration may imply that mechanisms other than apoptosis (e.g. necrosis) are involved.

We observed an heterogeneous response of Co_3_O_4_ and TiO_2_ NP on lipid peroxidation, a footprint of oxidative stress, and on IL-1β, TNF-α and MCP-1 gene expression, readouts of activation of inflammatory pathways. Co_3_O_4_, but not TiO_2_ NPs, strongly enhanced oxidative stress in CACs. TiO_2_ NPs uniformly up-regulated the expression of all three cytokines whereas Co_3_O_4_ NPs up-regulated IL-1β and TNF-α, but down-regulated MCP-1 expression. This might also suggests that Co ions are both bioaccessible and bioavailable after exposure to NPs in accordance with the evidence that Co causes oxidative stress [38]. A detailed EPR (electron paramagnetic resonance) study revealed that, in the presence of superoxide dismutase, cobalt in suspension (cobalt metal particles) is able to react with dissolved oxygen to generate ●OH [39].

Increased oxidative stress may tentatively be involved in reduced CAC adhesion [40,41] caused by Co_3_O_4_, but not by TiO_2_ NPs, for which other explanations need to be sought.

The lack of a stimulatory effect of the latter on oxidative stress in CACs is in apparent contrast with recent findings reporting reactive oxygen species activation in endothelial cells following incubation with TiO_2_ NPs [15,16] and with the widespread use of TiO_2_ particles, based on the diffuse belief that titanium is a biologically inert metal. These discrepant findings may be reconciled by considering that different cells were studied and that a relatively low concentration (20μg/ml) of TiO_2_ NPs was tested in our work.

The vivacious pro-inflammatory response of CACs to TiO_2_ is in agreement with observations in HUVEC, vascular endothelium and human alveolar and bronchial epithelial cells [15,16,42], and in animal models [43–45] whereas our observations with Co_3_O_4_ are almost unprecedented [46]. It should be emphasized that the pro-inflammatory effects of TiO_2_ were evident even at 1 μg/ml, the lowest concentration tested in our study.

With regard to the key process of endothelial replacement/repair involving CAC adhesion, the role played by inflammatory pathways is still unclear [47–50] with the possibility that, at some earlier steps, activation of distinct inflammatory pathways may be required for the process of endothelial replacement to occur. However, it may be speculated that early inflammation signalling in CACs triggered by exposure to metallic elements, especially TiO_2_ NPs, may play an untoward role both in the early steps of atherogenesis and in the biology of advanced/vulnerable plaques.

As a general remark, our observations in CACs should not be applied to other cell types as it is known that metal NP effects are cell-specific and strictly dependent on particle composition and physical properties, both critical determinants of the degree of cytotoxicity and of potential mechanisms involved [5]. An important property is represented by particle size, which is inversely related to biological activity of NPs: smaller NPs show a greater surface area per mass unit and an increased potential for biological interaction [51]. Accordingly, Co_3_O_4_ NPs which showed a higher cytotoxic effect displayed smaller dimensions than TiO_2_ NPs, although in our study the effect of NP physical and chemical properties on CACs was not investigated. To this aim further studies should use other available nano or sub-micro particles with the same chemical composition of the NPs used in this study, but with completely different physical and chemical properties. In addition, recent evidence showed that NP photoactivation enhances TiO_2_ toxicity [52], although in our study this aspect was not addressed.

## Conclusion

In conclusion, we have provided evidence that both TiO_2_ and Co_3_O_4_ NPs, albeit with different biologic profile, negatively affected CAC viability, morphology and function *in vitro*. Alterations in apoptosis, oxidative stress and inflammatory pathways may be involved, but further and more detailed mechanistic studies are needed to elucidate the molecular mechanisms underlying the observed alterations in CAC function/viability. TiO_2_ and Co_3_O_4_ NPs may affect CAC biology, thus potentially contributing to the increased risk of CV disease associated with nanosized NP exposure.

## Supporting Information

S1 Text Detailed method about structural and morphological characterization of TiO_2_ and Co_3_O_4_ NPs and determination of anatase and rutile proportion in TiO2 powder. (DOCX)

S1 Table Raw data. (XLSX)
