# Supplementary material for: Effects of TiO2 and Co3O4 Nanoparticles on Circulating Angiogenic Cells
Source: PLoS One. 2015 Mar 24;10(3):e0119310. doi: 10.1371/journal.pone.0119310 (PMC4372399; doi:10.1371/journal.pone.0119310)
Supplement: S1 Text — (DOCX) [file pone.0119310.s001.docx]

**Supporting Information**

**Methods**

Structural and morphological characterization

Structural and morphological characterization of NPs were performed by Transmission Electron Microscopy (TEM) on a 200 kV analytical JEOL JEM 2200-FS and subsequently by both Dynamic Light Scattering (DLS) and Z-potential techniques (90Plus PALS instrument by Brookhaven Corporation). Specimens for TEM analyses arose from deposition of one drop of a NP colloidal suspension in water (0.1mg/mL) on a TEM-grid, following 10 min of ultra-sonication. More diluted colloidal suspensions of the NPs in EGM-2 (0.01mg/mL) were dedicated to optical characterizations, following 10 min of ultra-sonication. To estimate the Stokes-Einstein radius of NP agglomerates in suspension, the fitting autocorrelation function with a minimization by non-negative least-squeares (NNLS) was used assuming a lognormal distribution of relaxation times.

At TEM analyses TiO_2_ NPs showed a regular spherical shape and appear as slightly aggregated. The size distribution is wide (std =0.57) and centered around the value of 38 nm, whereas Co_3_O_4_ NPs reveal an irregular not spherical shape, with tendency to form agglomerates of tens of NPs (data not shown). Co3O4 NP size distribution is narrow and centered around a mean value of 17 nm (data not shown). TiO_2_ NPs NP are polycrystalline, with a prevalent typical lattice spacing of the anatase structure, whereas Co3O4 NP are crystalline, with the typical inverse spinel crystal structure of cobalt ferrites.

The estimated specific surface areas are 13.8 m^2^/gr for TiO2 NPs and 46.7 m^2^/gr for Co_3_O_4_. Co_3_O_4_ NPs had a higher tendency to clusterize than those of TiO_2_. For Co_3_O_4_ NPs, Rh distribution was centered around 220 nm in H_2_O and 120 nm in EBM-2 (data not shown). With TiO_2_ NPs, the shift was from about 100 nm to 120 nm, with a modest aggregating effect of EBM-2 (data not shown). Z-potential values, reported in table 1, showed that the effect of EGM-2 is to reduce the surface charge of the NPs, probably due to the coverage of NPs by charged molecules present in the medium.

Determination of anatase and rutile proportion in TiO_2_ powder

Commercial TiO_2_ NP products are a mixture of different TiO_2_ polymorphs, characterized here by Raman spectroscopy (Horiba Jobin-Yvon Labram micro-Raman apparatus, equipped with a Olympus BH-4 confocal microscope and a 20 mW He-Ne laser emitting at 632.8 nm). A calibration curve was prepared to determine the anatase amount in the TiO_2_ nanopowder by collecting Raman spectra of different mixtures of anatase and rutile laboratory references (XRD tested) from 100wt% anatase to 100wt% rutile. Different ratios R of the areas (A) of selected anatase and rutile Raman bands (R_516/445_=A_516_/(A_516_+A_445_); R_143/445_=A_143_/(A_143_+A_445_); R_143/609_=A_143_/(A_143_+A_609_); R_516/609_=A_516_/(A_516_+A_609_) were determined by a peak-fitting procedure, as a function of the anatase content x (wt%). The function *y=ax/(ax+100-x)* was used to fit the data, *a*$\text{ }$being a fitting parameter, taken as the ratio of the Raman absolute intensities of anatase and rutile selected bands. The fitting curves, with a goodness of fit parameter *R*^2^~0.99, were used to determine the *x* value for the TiO_2_-NPs in the commercial product

Raman spectra collected on the TiO_2_ NPs display peaks corresponding to a mixture of anatase (tetragonal polymorph, space group I4_1_/amd, characterized by Raman peaks at~143-196-396-516-638 cm^-1^) and rutile (tetragonal polymorph, P4_2_/mnm, with~143-238-445-609 cm^-1^ characteristic Raman frequencies) ^[[1]](#endnote-1)^. All peaks for TiO_2_ NPs are broader than those of the pure polymorphs confirming the presence of nanosized (<100 nm) TiO_2_ particles ^[[2]](#endnote-2)^. The results of fitting curves indicated 93±1wt% anatase in the TiO_2_ powder.

1. Djaoued Y, Badilescu S, Ashrit P.V, Bersani D, Lottici P.P, Robichaud J. Study of anatase to rutile phase transition in nanocrystalline titania films, J. Sol-Gel Sci. Technol. 2002; 24, 255-264. [↑](#endnote-ref-1)
2. Bersani D., Lottici P.P., Ding X-Z. Phonon confinement effects in the Raman scattering by TiO2 nanocrystals, Appl. Phys. Lett. 1998; 72, 73-75. [↑](#endnote-ref-2)
